# Tics and Tourette Syndrome: A Literature Review of Etiological, Clinical, and Pathophysiological Aspects

**DOI:** 10.7759/cureus.28575

**Published:** 2022-08-30

**Authors:** Anshuta Ramteke, Yashwant Lamture

**Affiliations:** 1 Paediatrics, Jawaharlal Nehru Medical College, Datta Meghe Institute of Medical Sciences, Wardha, IND; 2 General Surgery, Jawaharlal Nehru Medical College, Datta Meghe Institute of Medical Sciences, Wardha, IND

**Keywords:** functional psychogenic tics, tiktok tics, deep brain stimulation, basal ganglia, immunological dysfunction, tic disorders, tourette syndrome

## Abstract

Tourette syndrome (TS) is a condition characterized by tics produced because of neuropsychiatric malfunctioning occurring in childhood, which becomes less severe in adulthood, followed by a difference in the severity of tics between two persons. TS is a diverse variable in which symptoms vary in different patients. It is associated with comorbidities like obsessive-compulsive disorder (OCD), attention deficit hyperactivity disorder (ADHD), and depression, and hampers the quality of life. Comorbid disorders must be investigated and treated as part of the clinical approach for all TS patients. Clinicians should be aware of the infrequent but serious neurological problems that can occur in these patients and recommend aggressively treating tics. Currently, there is more emphasis on symptom-based treatments by medicines, but as etiological knowledge improves, we will divert to disease-modifying medications in the future. Behavioral, pharmacological, and surgical methods can treat TS. Neuroleptics, other drugs, and behavioral therapies are the first-line options. Deep brain stimulation is evolving but has its pros and cons. The main focus of this review is on tics characteristics, how to manage and assess them, and limitations in the clinical spectrum.

## Introduction and background

Gilles de la Tourette Syndrome (TS) is a neurodevelopmental motor condition of childhood characterized by motor and vocal tics, first described in 1885 by French neurobiologist Georges Gilles de la Tourette [1]. The fifth edition of the Diagnostic and Statistical Manual of Mental Disorders (DSM-5) has classified tic disorders into three categories: Tourette’s syndrome, persistent motor or vocal tic disorder, and provisional tic disorder. Individuals with these illnesses all have tics that are described as non-rhythmic, abrupt, quick motor actions or vocalizations that occur repeatedly and are not caused by another disorder and are usually preceded by urges. For example, individuals might experience the impulse of clapping their hands impulsively and constantly, making faces/frowns or grunting, or even doing obscure actions such as waggling tongue movements. Although these actions might be appropriate in certain situations, the fact that they are repeated even in inappropriate cases is why they are considered abnormal [2]. Individuals can be classified into the type of tic disorder they belong to based on the following criteria: the number of motor or phonic/vocal tics, duration of tics, and age of the patient when tics first appeared.

Table 1 depicts that individuals with TS have numerous motor tics and not less than one vocal tic, but they need not necessarily occur together. The fact that both are present is noteworthy. Individual tics might change in incidence over periods, but they must persist for at least a year to be diagnosed as TS. Finally, in TS, the tics essentially start before the age of 18 years. Different studies show a male predominance of about 0.1-6%, and the overall prevalence rate of TS is 0.53% [3]. It is worth mentioning that nearly two-thirds of people diagnosed with TS have comorbidities, the most common being attention deficit hyperactivity disorder(ADHD) and obsessive-compulsive Disorder (OCD) [4]. Additional comorbidities often faced by an individual with TS are depression, disturbed sleep, emotional disorder, migraine, or other neuropsychiatric disturbances [4,5]. Tic disorders are most common prior to puberty, between four to six years of age, and severity is mostly in the age range of 10-12 years. The symptoms usually decrease in severity later as age progresses. Patients can deal with their symptoms through pharmacological or nonpharmacological treatments on a daily basis [6].

**Table 1:** Diagnosis of Tourette syndrome according to DSM-5 DSM-5: Diagnostic and Statistical Manual of Mental Disorders, Fifth Edition

TOURETTE SYNDROME
≥2 motor tics and ≥1 vocal tic
Persists for ≥ 1 year
Started before the age of 18

Although the neurobiology of TS is still incompletely understood, a lot of studies indicate that the caudate, putamen, globus pallidus, substantia nigra, and subthalamic nuclei, which constitute the basal ganglia have an important role. The basal ganglia are hypothesized to be involved in suppressing unwanted action apart from other diverse brain functions, which is why they are especially relevant to TS [7,8]. The principal excitatory neurotransmitter dopamine from the corticostriatal-thalamocortical circuit has been linked to the pathogenesis of TS [9,10]. Some studies mention the increased binding of dopamine to the D2 receptor in the caudate nucleus, which results in dopaminergic system dysfunction in TS patients [11]. However, the cause of TS is quite complex. Current studies have suggested one's neurobiological vulnerability to TS with multifactorial etiology such as genetic, environmental, and immunological factors [12]. The largest signal identified in a large genome-wide association study came from the gene COL27A1, which has rs7868992 on chromosome 9q32 but remains unclear [13]. A piece of rare stronger evidence for causing TS has been found due to histidine decarboxylase deficiency caused by gene mutation [14]. Educating the families of pediatric patients about the disorder's natural history can assist them in making treatment decisions. To this end, we will briefly examine the major findings concerning TS in several aspects.

## Review

**Figure 1:**
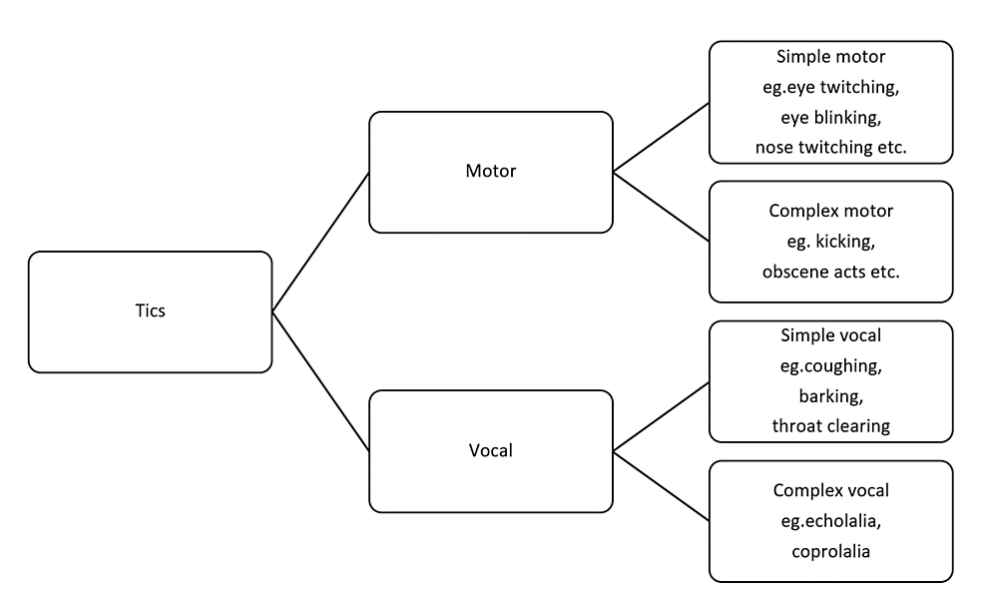
Classification of tics Image credit: Anshuta Ramtake

Tics could be classified into simple or complex, as depicted in Figure 1. Simple tics are often minimal in duration, spanning milliseconds, and can involve motor movements such as eye blinks or verbal habits such as throat clearing. Complex tics are frequently a mixture of simple tics, such as shaking one's head while shrugging their shoulders and persisting longer, sometimes over a second. Complex motor tics can include echopraxia, a tic-like repetition of other people's movements, and copropraxia, tics involving inappropriate comments. They can consist of echolalia (repeating the last word or phrase heard from others), palilalia (repeating one's own words or phrases), and coprolalia (saying words or obscenities) [1].

Individuals may sometimes detect a unique emotion or urge that happens prior to the commencement of a tic, such as an itch before reaching for a scratch. Tics are also more common or severe during stress, excitement, or tiredness. TS and associated tic disorders have no cure, but they can be treated with a mix of therapy and medication [1]. Table 2 summarizes the diagnosis of tic disorders according to DSM-5.

**Table 2:** Diagnostic classification of tic disorders according to the DSM-5 DSM-5: Diagnostic and Statistical Manual of Mental Disorders, Fifth Edition

Tourette Syndrome	Persistent motor or vocal tic disorder	Provisional tic disorder
Both multiple motors and one or more vocal tics are present.	There is the presence of either one or more motor or vocal tics but not both of them together.	Presence of one or more motor tics and/or one or more vocal tics
Tics persisting for >1 year since onset and may wax and wane.	Tics persisting for >1 year since onset and may wax and wane	Tics lasting for <1 year since the onset
Started < 18 years of age	Started <18 years of age	Started <18 years of age
Not influenced by any substance or condition	Not influenced by any substance or condition.	Not influenced by substance or condition.
_	No significant history of Tourette	No significant history of Tourette's or persistent tic disorder

Etiology

Genetics

Over the last year, there have been numerous advancements in TS genetics, many of which have resulted from large-scale cooperation. Genetic factors influence TS; patients' relatives have a higher incidence of tics, OCD, and ADHD. Monozygotic twins have a high prevalence rate, whereas dizygotic twins do not [15]. Although segregation results confirmed the autosomal-dominant concept, researchers today prefer a polygenic model [16]. An additional hypothesis is a bilinear inheritance, whereby both paternal and maternal family members may have a history of tics and/or comorbidities [17].

Only a small amount of de-novo coding variations have been linked to TS in recent research, including WW and C2 domain containing 1 (WWC1), fibronectin 1 (FN1), cadherin EGF LAG seven-pass G-type receptor 3 (CELSR3) along with nipped-B-like (NIPBL) [18]. WWC1 regulates trafficking, cell polarity, and migratory action. The NIPBL gene plays a dynamic role in the meiosis of cells and also holds the expression of genes during maturation in the mouse central nervous system. Axon pathfinding and cell polarity are assessed by the CELSR3 gene. The FN1 gene regulates cell proliferation, motility, and adherence [19].

Ercan-Sencicek et al. discovered a functional mutation by examining a two-generation family in histidine decarboxylase (Hdc) for immunological disturbance in TS [20]. The Hdc gene is essential for histamine production, which causes increased tic-like behavior; for example, excessive grooming was seen in Hdc mutant mice [21].

Environmental Risk Factors

Cesarean section, abnormal fetal growth, breech baby, and preterm birth were related to increased risk of TS. Thus, intrauterine and birth insults are risk factors [22]. Children who were given an antibiotic or hospitalized for infection were more prone to develop any psychiatric disease later in life. Surprisingly, tic disorders were the most likely to require antibiotics, followed by OCD. The likelihood of hospitalization was higher for people with intellectual disabilities, whereas the second most common was tic disorders. The link does not prove the relation of infections with TS [23].

Pediatric Autoimmune Neuropsychiatric Disorders Associated with Streptococcal Infections (PANDAS) is the most common disorder by group A *Streptococcus* (GAS) in a child and in adults is acute pharyngitis, accounting for about 20-37% of all pediatric cases. It can act as a disease-altering agent or trigger factor in TS, according to clinical research [24]. The diagnosis of PANDAS depends on the following factors: (1) presence of tic symptoms or OCD, (2) onset before puberty, (3) intermittent symptoms or variable remission and relapse, (4) temporal relationship between tic symptoms onset and infection with GAS, (5) presence of other neurological abnormalities in which the commonest are hyperactivity or choreiform movements [25].

In a population-based Taiwanese statewide retrospective investigation, Wang et al. showed that GAS infection causes higher chances of TS and ADHD. Another population-based study in the United States found that individuals who had a previous streptococcal infection were prone to TS, OCD, or tic disorder prior to initiation of symptoms. Furthermore, persons who have recently had repeated GAS infections are at a higher risk of developing TS [26]. GAS is not the sole pathogen involved in the genesis of TS. Enterovirus (EV), *Toxoplasma gondii*, *Borrelia burgdorferi*, *Mycoplasma pneumoniae*, *Chlamydia pneumoniae*, and even HIV have all been identified as pathogens [27].

**Figure 2:**
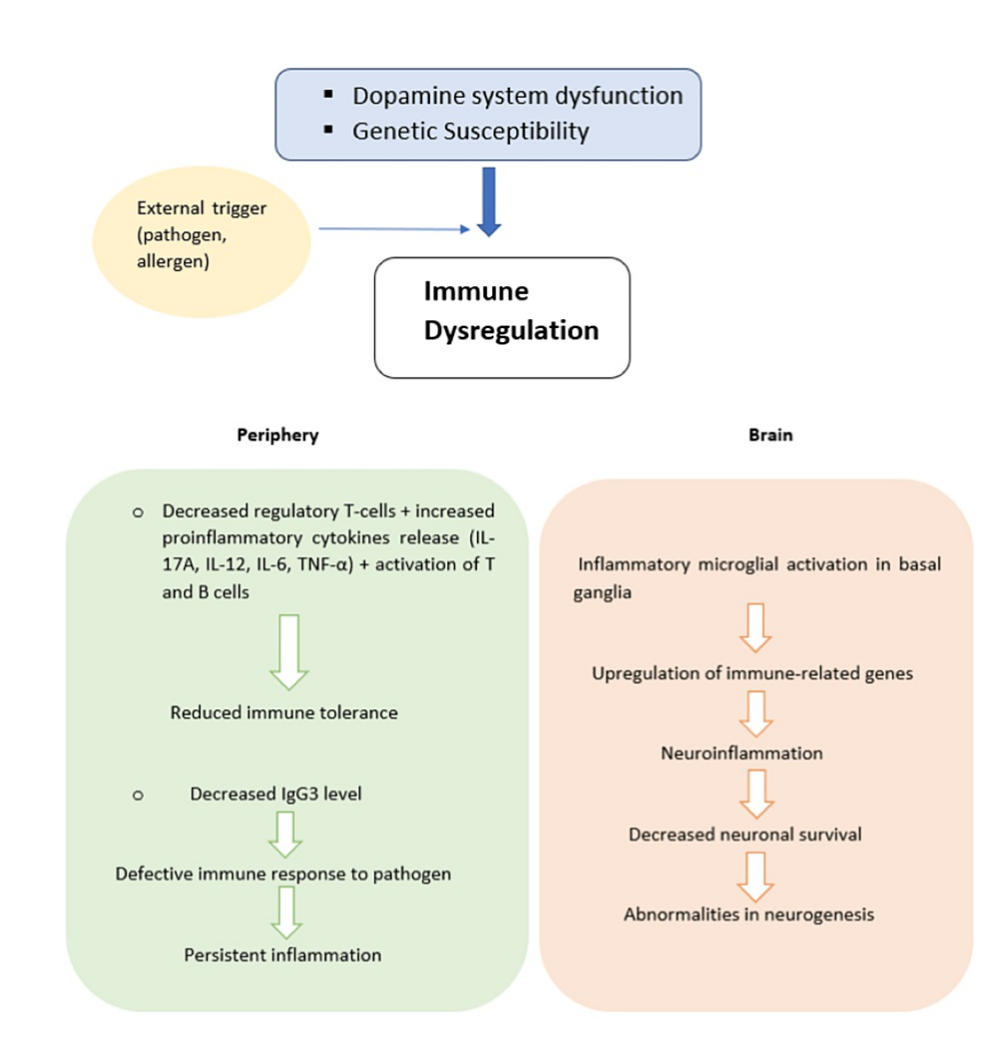
Immunological dysregulation in TS TS: Tourette syndrome; IL: interleukin Image credit: Anshuta Ramtake

Immunological Dysregulation in TS: Autoimmune disorders and allergies can both be caused by a breakdown in the immune tolerance process. Population-based studies were used in clinical reports that related allergy disorders to TS as summarized in Figure 2 [28].

Effects of Coronavirus Disease 2019 (COVID-19) Pandemic: Stress can also exacerbate or trigger tics which was observed during the period of the COVID-19 pandemic [29]. It was observed that the content of TS during COVID-19 increased on the social media site TikTok (ByteDance Ltd, Beijing, China) and was highly viewed by teenage girls, which resulted in portraying tic-like behaviors. This is an example of mass sociogenic illness. The simulation of tics viewed by the girls can be called as functional psychogenic tics [30].

The average age of onset of psychogenic movement disorder was 29.7 years. These patients had co-occurrence of other functional movement disorders and were unable to momentarily suppress movements; there was absence of premonitory sensations and presence of pseudo seizures. The difference between patients with TS and psychogenic tics is that the latter is common in older individuals, females are more affected than males, and there is no evidence of childhood or family history of tic disorder [31].

Pathophysiology

Structural Neuroimaging

Many neuroimaging studies have been done to know the affected part of the brain in TS patients, which in some studies revealed no difference in grey or white matter [32]. However, in other studies, it was discovered that there is a decreased thickness of grey matter and lower depth in internal, superior, and inferior, including pre and post-central frontal sulci [33]. In a prospective longitudinal study by Bloch et al., it was found that caudate volume in early childhood has a significant and inverse relation to the severity of tics [34]. With the help of voxel-based morphometry (VBM), there was evidence of a grey matter increase in the ventral putamen, left hippocampus, and midbrain. Connectivity reduction between basal ganglia and supplementary motor areas (SMA), along with frontal cortico-cortical circuits, was established with probabilistic fibre tractography [7].

Functional Neuroimaging

.Fluorodeoxyglucose (FDG) and positron emission tomography (PET) scans found two patterns, which include increased cerebral activity and bilateral premotor cortex along with metabolic activity decrease in the orbital frontal cortex and caudate/putamen [35]. With the help of flumazenil, a GABA receptor ligand, it was found that there is decreased binding in the bilateral thalamus, right insula, bilateral ventral striatum, and bilateral amygdala of TS patients and increased binding in bilateral substantia nigra, bilateral cerebellum, dentate nuclei, and right posterior cingulate cortex. This concluded that there is the involvement of the GABA-ergic system, which causes inhibitory loss in the brain of TS patients causing triggered rapid movements [36].

Involvement of the right dorsal anterior insula in the urge of tic had evidence as it is thought to influence cortico-striato-thalamic regions by not suppressing the urge of tic, which it normally does [37]. A study based on voxel-morphometry showed the involvement of the anterior dorsal region in a premonitory urge to tic and the posterior region in the generation of motor tics [38].

Neurophysiology

**Figure 3:**
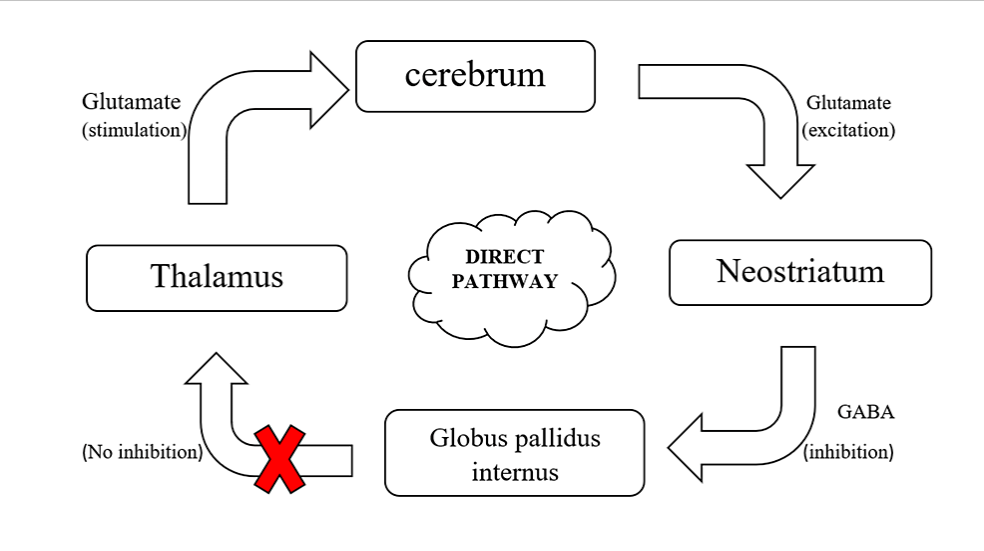
Mechanism of action of basal ganglia via direct pathway While glutamate is excitatory, gamma aminobutyric acid (GABA) is inhibitory [39] Image credit: Anshuta Ramteke

**Figure 4:**
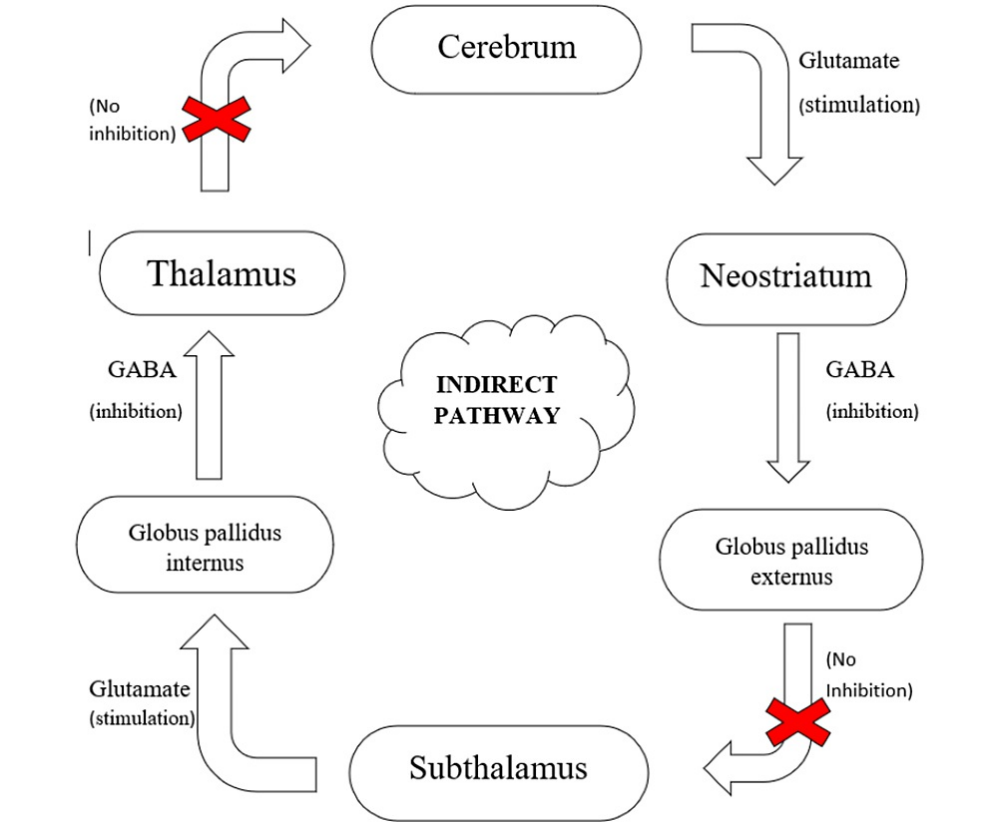
Mechanism of action of basal ganglia via indirect pathway While glutamate is excitatory, gamma-aminobutyric acid (GABA) is inhibitory [39] Image credit: Anshuta Ramteke

Basal ganglia have their function in the planning and programming of motor movements, suppression of both voluntary and involuntary movements, and cognition. It has two pathways: the direct pathway, which stimulates activities, and the indirect pathway, which inhibits actions, as described in Figures 3, 4 [39]. In individuals with TS, it's hypothesized that the faulty inhibitory mechanism in basal ganglia fails to stop unwanted signals from reaching the motor cortex (cerebrum). This causes the execution of undesired actions by the patient, which forms the basis of tics. It is thought that there is coupled reaction of failed inhibition in basal ganglia and increased activity in the motor pathway that results in the generation of movements [40].

**Figure 5:**
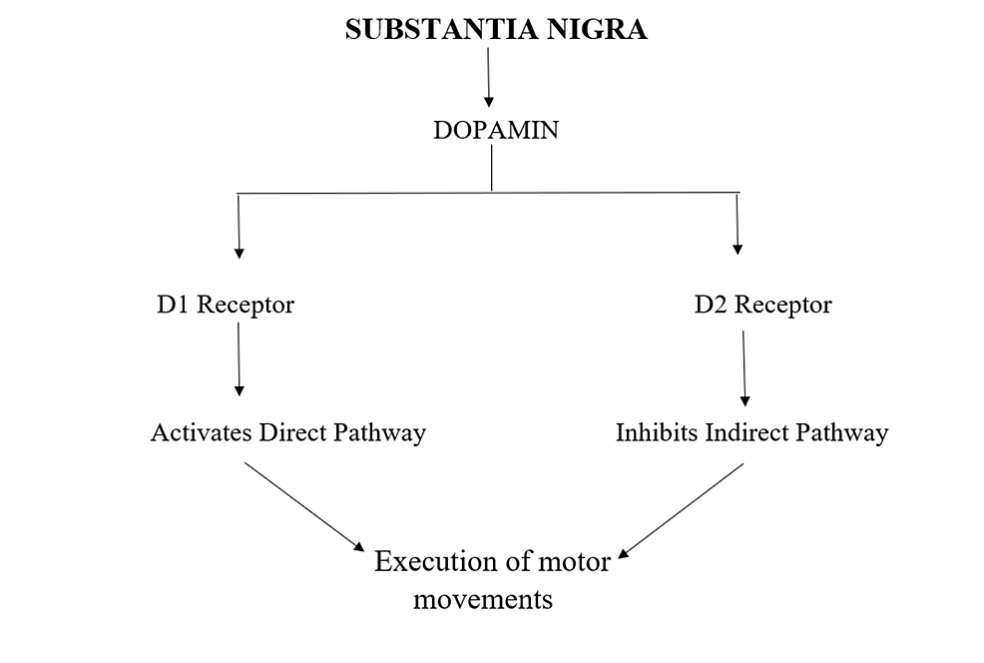
Mechanism of action of dopamine Image credit: Anshuta Ramteke

There is strong evidence that overactivity in the dopaminergic system is related to the generation of tic. Studies suggest that dopamine system hypersensitivity is due to developmental dysfunction in dopamine neurons. Dopamine is thought to send signals to relieve an urge to make movements. Figure 5 depicts the mechanism of dopamine action [11]. 

Differential diagnosis

Dystonia, chorea, stereotypies, athetosis, myoclonus, synkinesis, tremors, and hemiballismus are TS tics resembling symptoms presented in Table 3 [41]. Complex motor tics can show resemblance with stereotypies and can be difficult to distinguish from some compulsive rituals [42]. Vocal tics are extremely unusual and can be used to exclude other neurological diseases. An exception to this is Huntington's disease, where there could be vocalizations and brief sniffing.

**Table 3:** Differential diagnosis of Tourette syndrome

Features	Movements	Associated illness
Abrupt, brief, purposeless, repetitive jerky and stereotyped movements or utterances, which are exacerbated by stress and suppressible during sleep	Tics	Tourette syndrome, transient or chronic tic disorder
Sustained or intermittent muscle contractions causing abnormal, often repetitive, movements, posture or both	Dystonia	Wilson’s disease, torticollis, idiopathic torsion dystonia
Rapid, non-rhythmic, random, non-stereotyped, and unsustained movement, often superimposed on a voluntary movement that flow randomly from one part of the body to another	Chorea	Cerebral palsy, kernicterus, Sydenham’s chorea, Lesch-Nyhan syndrome, hereditary chorea, normal in children less than 8 months old
Voluntary, purposeless, and repetitive	Stereotypes	Mental retardation, autism, a pervasive developmental disorder
Slow, spontaneous, irregular writing movements of hands, fingers, toes and feet	Athetosis	Perinatal asphyxia, kernicterus, choreoathetosis
Involuntary, sudden, brief, jerky movements that are focal, multifocal or generalized	Myoclonus	Metabolic encephalopathies, juvenile myoclonic epilepsy, Wilson’s disease, anxiety, hypoxia
Involuntary movements associated with the specific voluntary act	Synkinesis	Physiologic
Involuntary, rhythmic, oscillating and usually distal movements of low amplitude on both sides of an axis	Tremors	Parkinson’s disease, drugs, metabolic disturbances, essential tremors
Involuntary, sudden violent flinging movement of extremities	Hemiballismus	Tuberculomas, stroke, amyotrophic lateral sclerosis, traumatic brain injury, nonketotic hyperglycemia, vascular malformations, neoplasms, complications from HIV infection, demyelinating plaques

Comorbid conditions

There are various comorbidities associated with TS. ADHD affects 20-90% of people with TS [43]. ADHD is a complicated neurological disorder characterized by inattention and hyperactivity/impulsive behavior. ADHD pathogenesis is complex in patients with TS and includes neurobiological factors, genetic and environmental [44]. OCD affects 11-80% of people with TS [43]. Obsessions (intrusive thoughts) and compulsions (repetitive behavior) are features of OCD, which result in adaptive dysfunction and emotional maladjustment. OCD symptoms in TS patients may differ from those seen in persons with primary OCD. TS individuals, for example, have increased symmetric preoccupation, "just right" perception, and obsessional counting (arithmomania); on the other hand, individuals with pure OCD have increased urge for compulsive washing, contamination worries, and cleaning rituals. Some persons with TS feel compelled to do things they shouldn't have to, such as making nasty or personal remarks that are out of character, etc. This might take the form of a tic or a more complex behavioral response called non-obscene socially inappropriate behavior. Depression, sleep Issues, and migraines are some comorbidities associated with TS. More severe sequelae of TS include myelopathy of the cervix, herniation of cervical disk, compressive neuropathy, arterial dissection, and stroke [45].

Treatment

**Figure 6:**
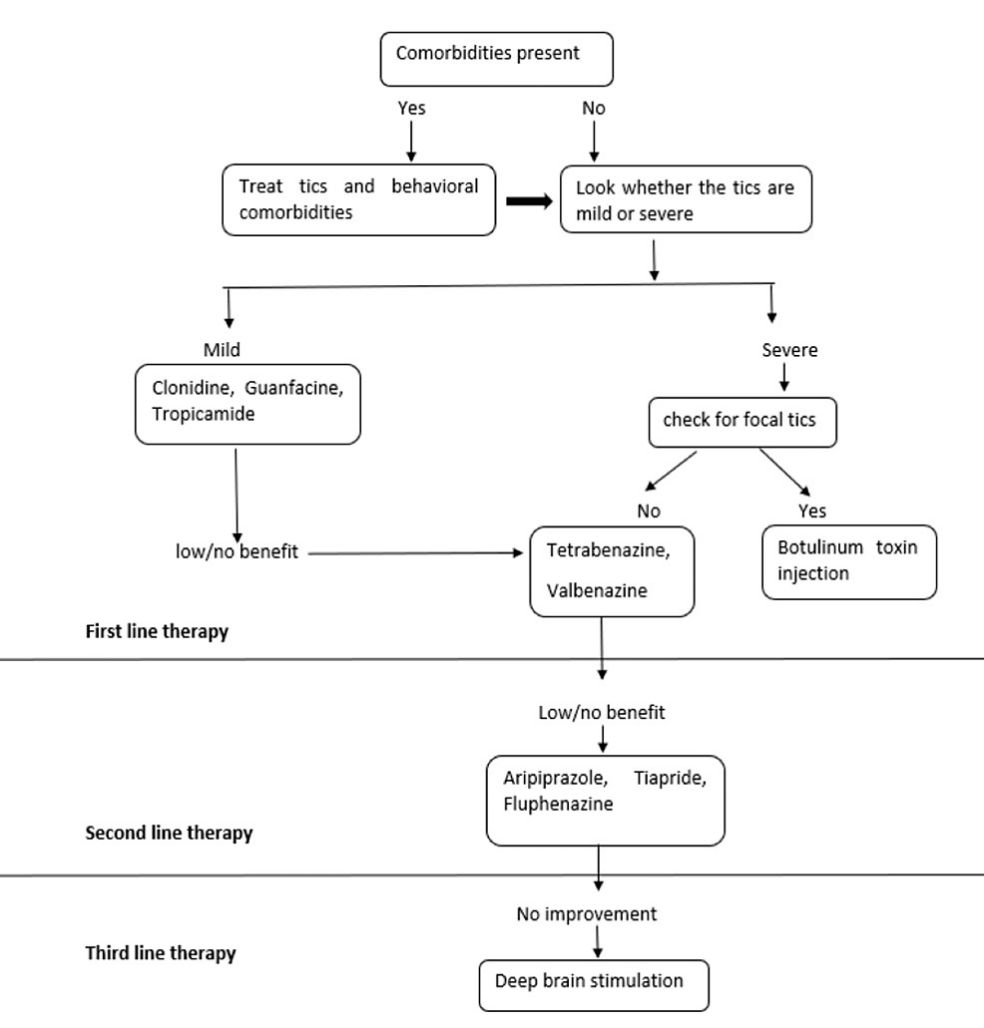
Summary for treatment of Tourette syndrome Image credit: Anshuta Ramteke

There are several approaches that can be employed to assist patients with unpleasant tics, summarized in Figure 6. Of course, the first careful thought is whether or not to treat because treatment is only symptomatic. Few individuals only experience minimal tics, so treatment could be more harmful than the disease. Furthermore, usually, tics are self-limiting and vanish on their own in many patients. However, if symptomatic treatment is required, effective therapy is available. The treatment has to be a multidisciplinary, individualized, and integrative approach. There are many ways in which one can assess the efficacy of any kind of therapeutic intervention, but by far, we rely on clinical grading scales like the Yale Global Tic Severity Scale (YGTSS), particularly the total tic severity component (TTS) [46]. Current Management includes behavioral, pharmacologic, and surgical treatments (Table 4).

**Table 4:** Treatment of Tourette syndrome

Name of treatment	Different options for that treatment
Behavioral therapy	Comprehensive behavioral intervention for tics (CBIT) and habitual reversal therapy
Alpha agonists	Clonidine, guanfacine
Dopamine receptor blockers	Fluphenazine, aripiprazole, haloperidol
Dopamine depleters	Tetrabenazine, valbenazine, deutetrabenazine
Antiepileptics	Topiramate
Botulinum toxin	Injection botulinum toxin
Deep brain stimulation	Thalamus, globus pallidus interna

Behavioral Treatment

All patients should be educated about the disease and, if possible, receive behavioral therapy for tics and/or comorbidities. Cognitive-behavioral therapies have an extended history along with excellent confirmation about two specific approaches. Comprehensive behavioral intervention is one of the methods. It is based on the habit-retraining therapy viewpoint, where the patient withstands the tic urge by producing any muscle motion that avoids the tic from occurring. Response prevention and exposure are other treatments where patients are taught to endure the urge to tic but refrain from doing so. Because motivation, learning difficulty, and other comorbidities can interfere with these treatments, they are not appropriate for all individuals. The availability of specialized clinical psychologists is the greatest barrier to treatment [47].

Pharmacological Treatment

Pharmacological medications like clonidine and guanfacine, vesicular monoamine transport type 2 inhibitors, topiramate, and tetrabenazine are often employed as first-line therapy for patients with tics who cannot be managed with behavioral therapy or when it is not accessible or available [48]. Antipsychotics such as aripiprazole, ziprasidone, risperidone, and fluphenazine are used as second-line therapy. Clonazepam (benzodiazepine) can be helpful but not be used as a first-line drug. These drugs are often effective, but they come with the risk of tardive dyskinesia and metabolic syndrome, along with some side effects [47].

Another possibility is a botulinum neurotoxin injection [49]. Botulinum toxin can be used to treat focal tics, particularly those involving the neck or eyes, as well as injections of the vocal cord to treat coprolalia and vocal tics, which are often accompanied by a hoarse voice. There is inadequate verification for using cannabis-derived substances like nabiximols, nabilone, and cannabidiol to treat tics. These drugs' most common adverse effects include dizziness, fatigue, and dry mouth. More research is needed before cannabis-based drugs may be properly prescribed to TS sufferers [50].

Although all existing dopamine receptor-blocking medications predominantly antagonize D2 receptors, there may also be a positive effect of D1 receptors inhibition. Ecopipam (D1 receptor antagonist) was developed initially as a capable antipsychotic medication in the 1980s, but it failed in schizophrenia trials. However, it has shown potential in treating tics [51].

Surgical Treatment

Deep brain stimulation (DBS) could be an alternate therapy option for severe and resistant TS sufferers. Patient selection, clinical assessment, risks, benefits evaluation, including mental comorbidities, selection of a target for DBS, treatment effectiveness, and clinical outcome selection are all important aspects of DBS treatment.

DBS in combination with radiosurgery is a potential technique for improving clinical outcomes in individuals with severe psychiatric comorbidities and needs to be carefully selected. Variances in the clinical response of individual patients for TS DBS have significance, and no predictor of personal responsibility has been established. Clinicians encounter a variety of ethical difficulties while doing DBS on pediatric patients. The clinical significance of the outcome of conventional open-loop DBS on TS symptoms has been established. Still, there could be a significant advancement in treating TS due to newly developed closed-loop DBS by adjusting stimulation as early as possible based on the patient's clinical state and the underlying pathology. Finally, TS DBS conduction must not be done without involving a multidisciplinary experienced team [52,53].

## Conclusions

TS is a complicated psycho-neurological disorder including motor and vocal tics and various additional comorbid conditions, which includes ADHD, OCD, depression, sleep issues, impulsive behavior, migraine, rage attacks, myelopathy of the cervix, and also dissection and stroke due to violent motor tics. The tics can be mildly or moderately bothersome, and in some circumstances, they can lead to self-harm or become otherwise debilitating. Comorbid cognitive and psychiatric problems can exacerbate overall impairment and reduce the quality of life. Based on clinical similarities, we believe that TS produced by genetic origin and Tourette-like syndrome (or secondary Touretism) induced by environmental causes are related to medical disorders with numerous etiologies. Education of the patient and a personalized and targeted therapeutic strategy are essential in its treatment. As a result, there is a requirement for a multifaceted approach, addressing motor symptoms as well as psychological/behavioral problems linked to TS DBS restriction, as it comes with its own set of dangers.

The body of knowledge about TS is rapidly expanding. However, a few easy yet critical questions are yet to be answered: Why do tics develop in children in the age group of 5-10 years? Why are they more prevalent among boys? Why do they decrease during sleep? Why do tics typically resolve as age progresses? How well can we forecast the prognosis of a single patient? Is secondary prevention a viable option? Hopefully, future research will address these and other critical challenges.
